# Towards Fixing Vessel Segmentation Breakage: A Systematic Review

**DOI:** 10.3390/jcm15156077

**Published:** 2026-08-05

**Authors:** Sébastien Goffart, Hervé Delingette, Andrea Chierici, Bernhard Föllmer, Amel Bakhouche, Jia Guo, Fabien Lareyre, Juliette Raffort

**Affiliations:** 1 Epione Team, Inria, Université Côte d’Azur, Sophia Antipolis, 06000 Nice, France; 2 University Hospital of Nice, 06003 Nice, France; 3 Fédération Hospitalo-Universitaire FHU Plan&Go, 06003 Nice, France; 4 Department of Digestive Surgery, University Hospital of Nice, 06003 Nice, France; 5 Laboratory of Molecular Physio Medicine (LP2M), UMR 7370, CNRS, University Côte d’Azur, 06003 Nice, France; 6 Department of Vascular Surgery, Hospital of Antibes-Juan-les-Pins, 06600 Antibes, France; 7 Clinical Chemistry Laboratory, University Hospital of Nice, 06003 Nice, France

**Keywords:** segmentation, vessel discontinuity, topology, post-processing, CTA, vascular reconstruction

## Abstract

**Background/Objectives**: Accurate arterial tree reconstruction from computed tomography angiography (CTA) is essential for vascular diagnosis, surgical planning, and hemodynamic modelling. A persistent and underappreciated problem is vessel discontinuity: thin distal branches appear as disconnected fragments rather than continuous structures, caused by bifurcations, image noise, contrast variation, arterial plaque, motion artifacts, and partial volume effects. This scoping review aimed to systematically characterize computational approaches addressing vessel breakage in CTA segmentation and identify methodological gaps warranting further investigation. **Methods**: This scoping review was conducted in accordance with PRISMA-ScR guidelines. Google Scholar and PubMed were queried for studies published between March 2000 and March 2026. Eligible studies included peer-reviewed journal articles and conference proceedings presenting original methodological contributions to three-dimensional vascular segmentation from CTA. **Results**: Three generations of computational solutions were identified: classical geometric and geodesic methods, deep learning approaches with topology-aware training, and hybrid post-processing frameworks. Topology-sensitive metrics (clDice, Topology Sensitivity) were identified as preferred metrics to better capture clinical utility than standard voxel-based metrics such as the Dice coefficient. **Conclusions**: Vessel discontinuity remains a clinically relevant and challenge in vascular CTA segmentation. Hybrid post-processing frameworks combining deep learning with geodesic connectivity restoration represent the current state of the art. Standardized adoption of topology-aware evaluation metrics is recommended to better reflect clinical utility in future studies.

## 1. Introduction

Computed tomography angiography (CTA) plays a central role in the clinical assessment of vascular disease, supporting diagnosis, treatment selection, procedural planning, and computational modelling. Reliable vessel segmentation enables a wide range of downstream applications, including stenosis quantification, calcium scoring, three-dimensional vascular reconstruction, and patient-specific haemodynamic simulations [1,2]. In clinical practice, the usefulness of these reconstructions depends not only on their spatial accuracy but also on their ability to reproduce the continuity and branching pattern of the arterial tree.

A persistent yet under-recognised limitation of current segmentation methods is vessel breakage, whereby anatomically continuous vessels are reconstructed as fragmented and disconnected segments. This issue is particularly prevalent in small-calibre distal branches and in the presence of pathological alterations such as calcifications, severe stenoses, motion artefacts, low contrast opacification, image noise, and partial-volume effects [3,4]. Although such interruptions may involve only a limited number of voxels, they can substantially alter the anatomical interpretation of the reconstructed vessel.

The clinical consequences of vessel breakage may be relevant across several vascular territories. In peripheral artery disease (PAD), for example, the continuity of infrapopliteal vessels directly influences the assessment of distal run-off, the identification of potential revascularisation targets, and procedural planning [5,6]. An artificial interruption may make a patent but poorly opacified vessel appear occluded, obscure a distal target vessel, or prevent the extraction of a continuous centreline. More broadly, incomplete or fragmented segmentations can compromise haemodynamic simulations, bias quantitative imaging biomarkers, and limit the reliability of automated analysis pipelines [4,7]. This limitation is especially important because a segmentation may appear quantitatively accurate while remaining clinically or anatomically inconsistent. Conventional overlap-based evaluation mainly assesses whether the predicted vessel voxels correspond to the reference segmentation, but it may not adequately reflect whether the reconstructed arterial tree remains connected. Consequently, a small discontinuity may have only a limited effect on global segmentation performance while interrupting an entire vascular pathway.

From a methodological perspective, one contributing factor to vessel breakage is a mismatch between voxel-based optimization objectives and the topological nature of vascular anatomy. Conventional approaches, including classical image processing techniques [8] and deep learning models, are primarily designed to maximise local accuracy rather than enforce global structural consistency [9]. As a result, clinically relevant segmentation errors may manifest not only as incorrect voxel classification, but also as loss of connectivity, missing branches, or anatomically implausible interruptions. To address this limitation, topology-sensitive evaluation metrics have been proposed, including centerline, graph or connectivity-based measures, which explicitly assess structural continuity rather than relying solely on voxel overlap [10].

In this context, the present scoping review aims to systematically analyse computational approaches designed to address vessel breakage in CTA segmentation. Among the various vascular imaging modalities, CTA is one of the most commonly used in clinical practice, as recommended by current guidelines [6,11]. We focus specifically on methods that seek to preserve or restore vascular continuity, including topology-aware losses, graph-based networks, transformer-based models and hybrid post-processing frameworks. By synthesising current evidence, we aim to identify methodological trends, highlight persistent limitations, and outline key directions for future research toward reliable, topology-preserving vascular segmentation.

## 2. Materials and Methods

### 2.1. Literature Search Strategy

**Figure 1:**
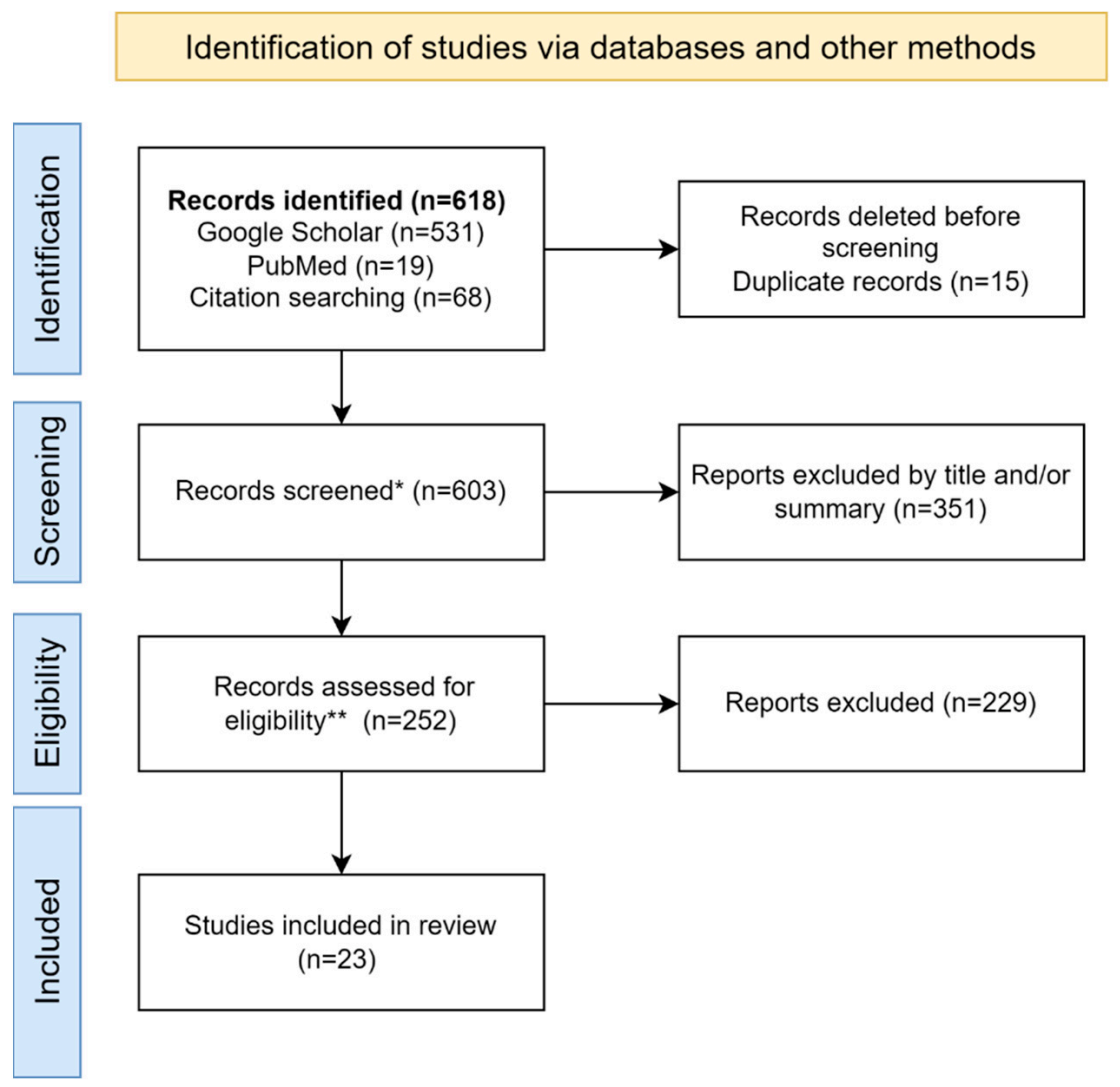
PRISMA flow chart diagram for selection of articles. * The report screened corresponds to a first sorting based on the inclusion criteria based on the title and the abstract content regarding the outcome and the format (journal article only). ** The report assessed for eligibility corresponds to a deeper analysis of the research publication content; all the extracted data are expected in the article.

A literature search was conducted in March 2026 using Google Scholar and PubMed to identify studies on vessel segmentation and reconstruction according to the Preferred Reporting Items for Systematic Reviews and Meta-Analyses (PRISMA) guidelines described in the flowchart (Figure 1) [12] and detailed in the Supplementary Table S1: PRISMA 2020 Checklist. Articles published between 1 March 2000, and 31 March 2026, were considered. The strategy combined the following terms in a query, which can be entered directly into the Google Scholar search bar: intitle: (vessel OR coronary) intitle: (segmentation OR extraction) (“CT angiography” OR CTA) (“topology-preserving” OR reconnection OR “gap filling” OR “centerline completion” OR “fully connected”) (“shortest path” OR geodesic OR tracking OR graph) (method OR framework OR algorithm OR model) (dataset OR experiments OR results) -intitle:review -intitle:survey -intitle:overview—“systematic review”—“meta-analysis”. Complementary, the PubMed query is: (vessel OR coronary OR Artery) AND (segmentation [Title] OR extraction [Title]) AND (“CT angiography” OR CTA) AND (topology OR reconnection OR “gap filling” OR continuity). The search was further supplemented by manual screening of reference lists of included articles and relevant reviews, enabling the identification of additional studies through cross-referencing during the review process. This systematic review was not registered in PROSPERO, as the registry is primarily intended for reviews addressing clinical or health-related outcomes, whereas the present work is technical in nature, focusing on computational segmentation methodologies rather than a health-related outcome.

### 2.2. Study Eligibility Criteria

The focus was on methods generating accurate and structurally consistent three-dimensional (3D) representations of vascular networks, including coronary, peripheral, and abdominal arteries, with demonstrated applicability to multiple anatomical vascular territories (e.g., validation on both coronary and peripheral datasets or explicitly reported by the authors). Only studies based on high-resolution CTA data, validated on human subjects, and published in English from 2000 onward were considered. Studies limited to two-dimensional analysis or relying on other imaging modalities (e.g., X-ray angiography, digital subtraction angiography, or C-arm CT) were excluded. Review articles and studies lacking methodological novelty were also excluded, although their references were screened for additional relevant works. The availability of an explicit algorithmic description was also mandatory (e.g., mathematical formulation, pseudocode, or detailed workflow) allowing understanding and potential reproducibility of the method. Studies without standardized quantitative evaluation were retained if they provided qualitative or structural validation of the proposed method. Study selection was performed by one reviewer, with a second reviewer validating the selected studies; disagreements were resolved by discussion.

### 2.3. Performance Metrics Limitations

The Dice similarity coefficient remains the dominant metric for evaluating vascular segmentation performance, yet it is fundamentally inadequate for assessing the clinical relevance of vessel reconstructions [13,14]. Dice quantifies voxel-level overlap between predicted and reference segmentations, rewarding spatial accuracy without regard for structural integrity. A segmentation in which a vessel has been severed at multiple points may achieve a Dice score nearly identical to a fully connected reconstruction, provided the disconnected fragments occupy approximately the same spatial positions as the true vessel. This insensitivity to topological errors is particularly problematic in the context of peripheral arterial disease, where the continuity of infrapopliteal vessels directly determines treatment planning and the feasibility of surgical or endovascular revascularisation.

Several topology-aware metrics have been proposed to address these limitations. The clDice (centreline Dice) coefficient evaluates overlap along the vessel skeleton rather than across the full voxel volume, simultaneously capturing the completeness of vessel tracing and the precision of centerline recovery, making it sensitive to breaks that Dice would not detect [10]. The ccDice metric extends this principle using a connected component framework, penalising segmentations that produce multiple disconnected fragments in place of a single continuous structure [9]. Beyond overlap-based measures, Topology Sensitivity (TS) quantifies what proportion of the true vascular tree was successfully recovered, directly penalising missing branches and occult disconnections, while Topology Precision (TP) assesses whether the predicted segmentation introduced phantom vessels or false connections not present in the reference. Despite their conceptual advantages, these topology-aware metrics remain largely confined to research settings and have not yet been widely validated or adopted in routine clinical practice. Reporting of these metrics was recorded where available in the included studies, but was not required for study inclusion.

## 3. Results

### 3.1. Study Characteristics

A total of 618 records were identified through database searching (Google Scholar, *n* = 531, PubMed, *n* = 19) and citation tracking (*n* = 68). After initial screening based on titles and abstracts, 15 duplicates were removed, 351 reports were excluded according to predefined inclusion criteria. The remaining 252 articles underwent full-text assessment for eligibility, of which 229 were excluded following detailed evaluation of study content and data availability. Ultimately, 23 studies were included in the final review. The study selection process is summarized in Figure 1. When available, at least one voxel-based metric and one topological metric were extracted and reported for each study. Metrics are limited to a minimal, consistent set and reported on public datasets whenever possible. Because studies used heterogeneous performance metrics, datasets, and reference standards, direct quantitative comparison across studies was not feasible; results are therefore synthesized narratively and grouped by methodological approach rather than ranked by performance.

### 3.2. Common Benchmark Datasets

Across the 23 included studies, coronary artery segmentation was by far the most represented anatomical territory (*n* = 12, 52%), followed by hepatic and aortic applications (*n* = 3 each), while head, neck and cerebral vasculature (*n* = 2), and pulmonary, abdominal, and multi-territory approaches (*n* = 1 each) were comparatively underrepresented, highlighting a strong research focus on coronary CTA segmentation relative to other vascular regions.

Several publicly available datasets have been adopted as benchmarks for three-dimensional CTA vessel segmentation across distinct anatomical territories. For coronary segmentation, the most widely used resources are the ASOCA dataset, which provides cardiac CTA with expert coronary artery annotations [3], and ImageCAS, a large-scale dataset specifically designed for coronary artery segmentation [15].

For hepatic segmentation, the IRCADb database offers annotated liver CT scans including hepatic vascular structures [16]. For abdominal multi-organ segmentation, LIDC-IDRI [17] and AMOS [18] provide multi-structure annotations enabling evaluation of segmentation models across abdominal vascular territories. For aortic segmentation, three dedicated benchmarks have been used: ImageTBAD, focused on type-B aortic dissection [19]; AVT, providing aortic vessel tree annotations [20]; and AortaSeg24, a recent multi-class aortic segmentation challenge dataset [21].

Among the 23 reviewed studies, ASOCA was the most frequently used public benchmark dataset (*n* = 7, 30%), followed by private institutional datasets (*n* = 7, 30%) and 3Dircadb (*n* = 3, 13%), while ImageCAS and AortaSeg24 were each used in 2 studies. Several other datasets (MM-WHS, ISICDM 2020, LIDC-IDRI, AMOS, AVT) were each used only once, reflecting a heavy reliance on a few public coronary and hepatic benchmarks, alongside a substantial proportion of non-reproducible private datasets.

### 3.3. Classical Geometric Enhancement

Hessian-based vesselness filters, of which the Frangi filter is the most widely adopted, represent the foundational classical approach to vascular segmentation [22,23]. These filters analyse local image curvature by computing the eigenvalues of the Hessian matrix at each voxel, producing a tubularity score that reflects how closely the local intensity structure resembles a cylindrical vessel. Their principal advantages are computational efficiency, interpretability, and independence from image resolution, properties that have made them a standard preprocessing step in clinical workstation tools and in hybrid pipelines combining vesselness enhancement with region-based segmentation, such as fuzzy connectedness approaches. For instance, Zhang et al. combined vesselness-enhanced images with fuzzy connectedness and adaptive filtering for hepatic vessel segmentation on the 3Dircadb dataset (*n* = 20), achieving high voxel-wise accuracy (Acc: 0.97) but comparatively modest Fuzzy Connectedness (FC: 0.74), illustrating that strong overlap-based performance does not necessarily reflect preserved vascular continuity [24].

However, Hessian-based filters operate at the voxel level and do not explicitly encode connectivity. As such, they cannot determine whether adjacent tubular regions belong to the same vessel or represent distinct, unrelated structures, and they provide no mechanism for detecting or localising vessel discontinuities. These limitations have motivated the development of additional geometric and topological strategies that explicitly model vessel connectivity, including graph-based reconstruction and persistence-driven ridge tracking approaches. Szymczak et al. proposed a persistence-based ridge detection method combined with minimum spanning tree (MST) reconstruction and geometric pruning, explicitly incorporating gap-filling and branch-trimming steps to restore coronary tree connectivity on a private dataset (*n* = 64); however, this approach was evaluated only qualitatively, limiting direct performance comparison with other geometric methods [25].

Geodesic and minimal-path methods address the problem of vessel tracing from a different perspective, reformulating it as a shortest-path optimisation problem [26]. Given two user-defined endpoints, these algorithms identify the lowest-cost route through the image volume, where cost is defined to be low in regions that are bright, tubular, and vessel-like, and high in surrounding tissue. The resulting path is mathematically optimal under the chosen cost function, and methods of this class underpin many semi-automatic tracing tools used in clinical annotation and image analysis workflows, often in combination with vesselness filtering [24]. Despite their theoretical elegance, these approaches carry significant practical limitations. They require manual seed placement at vessel endpoints, precluding fully automated application across complex vascular trees. They produce only a one-dimensional centreline path rather than a volumetric segmentation, and they provide no mechanism to detect automatically where vessel breakage has occurred. Furthermore, they cannot incorporate probabilistic confidence information from deep learning models, and their performance degrades in the presence of arterial calcification or local intensity ambiguity, both of which are common features of infrapopliteal CTA in patients with advanced peripheral arterial disease. Overall, vesselness-based methods reach high accuracy but only preserve connectivity implicitly, while ridge-tracking methods explicitly target continuity, though validated only qualitatively. Neither method can automatically detect vessel breakage, which motivates the topology-aware approaches discussed next.

### 3.4. Deep Learning Approaches

Deep learning-based approaches have been widely applied to vascular segmentation across multiple anatomical territories, including coronary, aortic, cerebral, abdominal, pulmonary, and head-and-neck vessels (Table 1). Several studies propose architectures that incorporate topology-aware learning. Most approaches build upon encoder–decoder architectures derived from U-Net, which remain the dominant baseline for volumetric vessel segmentation, especially the nnU-Net [27]. Shit et al. (2021) introduced the clDice loss, a differentiable topology-aware objective designed to preserve connectivity in tubular structures [10]. Chen et al. (2026) extended this concept with CLGeoDice, reporting Dice = 0.87 and clDice = 0.88 on a dataset of 160 coronary CTA scans [28]. Similarly, Acebes et al. (2024) proposed the centerline Cross-Entropy (cICE) loss, achieving Dice = 0.80 and clDice = 0.79 on the ASOCA dataset (*n* = 40) [29]. Graph-based and topology-driven architectures have also been explored. Guzzi et al. (2026) applied a Hausdorff distance loss for lower-limb arterial segmentation in patients with peripheral artery disease, achieving a clDice score of 0.88 on a private dataset [4]. Yao et al. (2023) introduced TaG-Net, a topology-aware graph network integrating voxel segmentation and centerline-based structural encoding, evaluated on 401 head and neck vessel cases [30]. Ozcelik et al. (2026) applied persistent homology constraints for aortic segmentation, reporting Precision = 0.88, Recall = 0.87, and F-score = 0.87 (*n* = 24) [31]. Zhao et al. (2025) proposed a morphology-guided framework with attention-based tracking, achieving 95% centerline overlap on the ImageTBAD dataset (*n* = 100) [32]. Other approaches focus on improving vessel representation and continuity. Wu et al. (2026) introduced FlowAxis, which models vessels using adaptive keypoints to preserve topology across coronary and cerebral datasets (ASOCA and ImageCAS-HNCTA) [15,33]. Liu et al. (2026) proposed TopoVST, combining multi-scale sphere graphs and wave-propagation tracking, achieving overlap = 0.87 on ASOCA and AortaSeg24 datasets [34]. Ye et al. (2025) presented VSR-Net, a curvature-clustering approach for reconstructing disrupted pulmonary vessels, reporting Dice = 0.89 and Jaccard = 0.76 on a dataset of 1000 cases [35]. Additional deep learning architectures target specific vascular territories. Liu et al. (2025) introduced DTUNet for cerebral vessel segmentation (*n* = 50) [36], while Epifanov et al. evaluated a hybrid U-Net and gradient-based approach on abdominal datasets (*n* = 142; LIDC-IDRI and AMOS) [37]. Iltaf et al. (2025) proposed VesselSAM, a SAM-based architecture with atrous attention for aortic segmentation, achieving Dice = 0.93 on AVT (*n* = 56) and TBAD (*n* = 100) [38].

Overall, these studies reflect three main trends: (1) most architectures remain built on U-Net/nnU-Net backbones, with topology-awareness introduced either via the loss function (clDice, cICE, persistent homology, Hausdorff-based losses) or via the architecture itself (graph neural networks, keypoint-based representations); (2) about half of the reviewed methods explicitly target vessel connectivity/breakage as their main objective [4,28,29,30,33,34,35], while the others primarily optimize overlap-based accuracy without an explicit connectivity mechanism [31,32,33,34,36,37,38]; and (3) evaluation metrics remain highly heterogeneous, combining overlap-based (Dice, Jaccard), distance-based (HD95, ASSD, ASCD), and topology-specific (clDice, Betti number, centerline overlap) measures, limiting cross-study comparability.

### 3.5. Hybrid & Post-Processing Refinement

Hybrid approaches integrate learned segmentation with explicit structural refinement strategies, such as graph-based reconnection, pre-processing including vesselness filtering, and centerline-based corrections, as summarised in Table 2. Several hybrid frameworks integrate deep learning with classical pre-processing or structural constraints. Survarachakan et al. (2021) proposed a pipeline combining vesselness filtering (Sato, Frangi), gamma correction, and feature fusion prior to CNN-based segmentation, evaluated on 57 hepatic CT scans and achieving Dice = 0.80 [39]. Similarly, Alirr et al. (2024) combined vesselness enhancement, region-of-interest extraction, and ResUNet segmentation for coronary CTA, reporting Dice = 0.87 anreporRecall = 0.88 on a combined dataset of 100 cases (ASOCA and MM-WHS) [40]. Park et al. (2026) introduced a hybrid method integrating U-Net segmentation with gradient-based contour detection, achieving Dice = 0.82 on ASOCA [41]. Other approaches focus on explicit structural reconstruction. Qiu et al. (2025) proposed CorSegRec, a graph-based post-processing method designed to reconnect disconnected vessel segments and enforce full arterial tree connectivity, evaluated on coronary datasets (ASOCA and PDSCA) [42]. Guo et al. (2020) introduced a multi-stage pipeline combining preprocessing, 3D graph cuts, thinning-based centerline extraction, and vascular completion, demonstrating reconstruction of complete hepatic vascular networks on the 3Dircadb dataset [43]. Classical geometric components remain integral to several hybrid pipelines. In parallel, implicit representation methods have been explored for geometric refinement. Esposito et al. (VesselSDF) model vascular structures using a neural signed distance function on Hepatic vessel dataset and IRCADb, enabling continuous surface representation and differentiable optimisation. Applied as a post-processing step, the method focuses on improving surface smoothness and correcting local boundary discontinuities [44]. Beyond structural refinement, hybrid methods have also addressed the challenge of disentangling overlapping vascular structures. Wang et al. (2024) proposed AVDNet, jointly segmenting coronary arteries and veins through a topology-based vessel refinement network (TVRN) combined with an inverse distance weighted Dice loss to recover thin branches, evaluated on ASOCA (Dice = 0.88, centerline recall = 3.6) and a private dataset of 700 patients [45]. Similarly, Liu et al. (2026) introduced Topo-UNet, a topology-aware multi-task network integrating a Bidirectional Slice-wise ConvLSTM module for spatial continuity and a Gaussian-based auxiliary task for fine vessel recovery in pulmonary CTA, achieving Dice = 0.91 and IoU = 0.83 on the ISICDM 2020 dataset [46].

These hybrid strategies can be grouped into four main categories: vesselness-based pre-processing [39,40,41], which enhances low-contrast vascular structures prior to CNN segmentation but does not explicitly enforce connectivity; graph-based reconstruction and reconnection methods [42,43], which act directly on vessel topology by reconnecting broken segments or completing missing branches; topology-aware refinement networks [45,46], which embed connectivity constraints within the learning process itself through dedicated modules or losses; and implicit representation approaches [44], which model the vessel surface continuously to improve smoothness and local boundary consistency. Compared with purely deep learning methods, hybrid approaches do not consistently yield higher Dice scores, but several reports improved connectivity-related outcomes (e.g., centerline recall, HD95, or qualitative completeness of the reconstructed vascular tree), indicating that their main added value lies in structural continuity rather than overlap accuracy. However, this improvement is not systematic across all hybrid methods: vesselness-based and implicit representation approaches primarily refine segmentation quality or surface smoothness without explicitly guaranteeing connectivity, whereas graph-based and topology-aware methods are specifically designed to address vessel breakage and show more consistent gains in connectivity-related metrics. Post-processing therefore improves vascular continuity mainly when connectivity is an explicit design objective, rather than as an automatic consequence of combining learning-based and classical components.

## 4. Discussion

**Figure 2:**
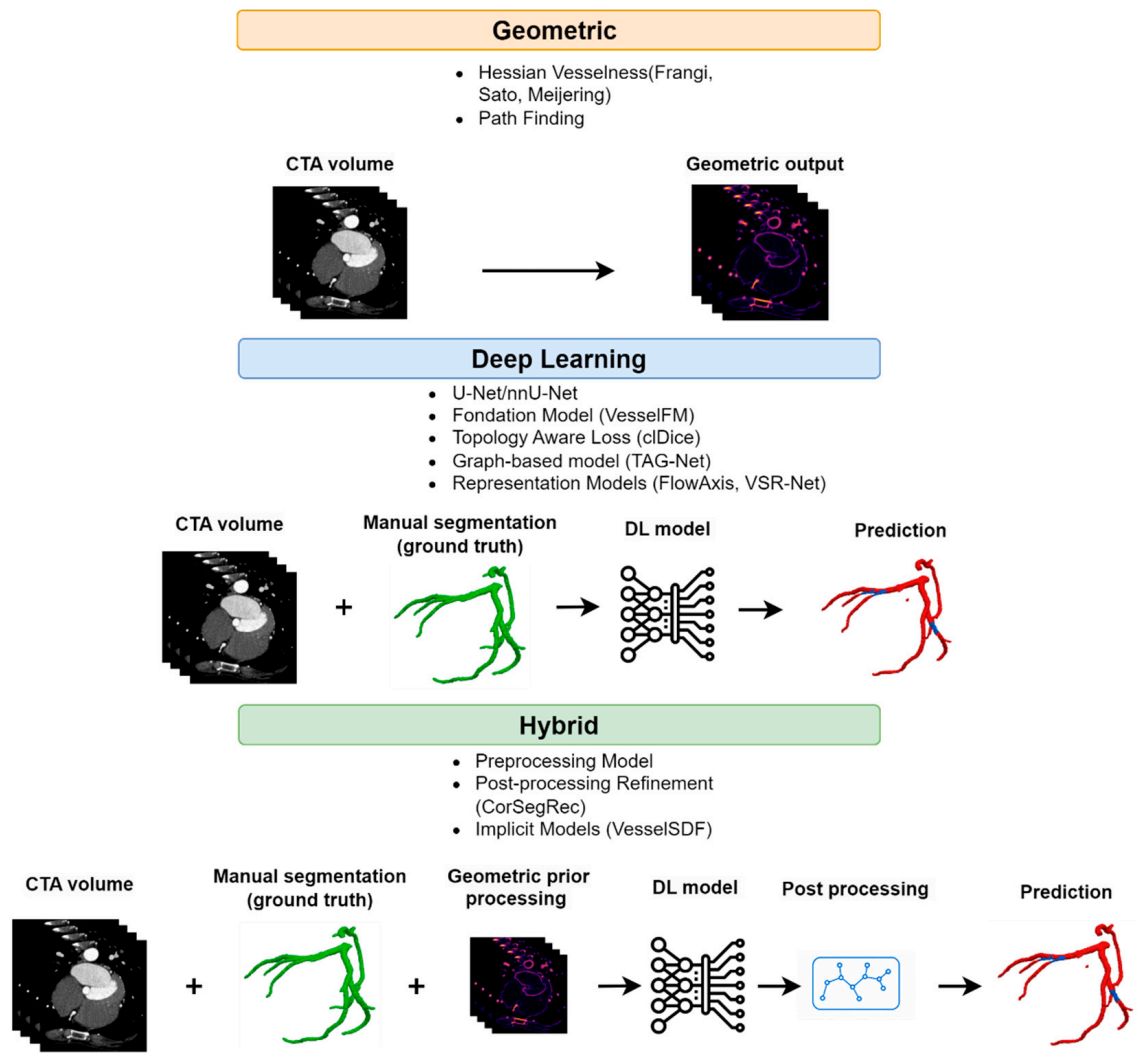
Summary of the methods grouped by Geometric, Deep Learning, Hybrid approaches.

The full results of the reviewed methods are summarised in Figure 2, which provides a structured overview of the three main methodological categories identified in this review. Overall, the reviewed literature reflects a consistent trade-off across categories: geometric methods offer interpretability and require no annotated data but lack global topological awareness; deep learning methods achieve strong overlap-based accuracy but frequently fail to preserve connectivity unless topology-aware mechanisms are explicitly included; and hybrid methods attempt to combine both paradigms, improving connectivity in some cases at the cost of additional pipeline complexity. Each category is characterised by distinct strategies for vessel extraction and connectivity preservation, along with specific strengths and limitations that are discussed in the following subsections.

The CTA modality was chosen as the focus of this review given its widespread clinical use and the abundance of dedicated segmentation methods reported in the literature. Nonetheless, vessel breakage remains a modality-agnostic issue also relevant to MRA and DSA.

### 4.1. Geometric Enhancement Methods

Geometric approaches provide a principled and interpretable framework for vessel extraction by explicitly modelling tubular structures through image-derived features. Their independence from training data enables deployment across heterogeneous datasets and avoids the need for extensive annotation, which is particularly advantageous in clinical contexts where labelled data are scarce. Geometric methods remain the preferred choice when annotated datasets are unavailable or when a fast, deployable solution is required without model training, such as in exploratory or single-center settings. However, these methods fundamentally rely on local image cues and therefore lack a global understanding of vascular topology. As a result, although vesselness filters enhance tubular structures, they do not inherently guarantee structural continuity, especially in the presence of calcifications, severe stenoses, or intensity inhomogeneities. This limitation is corroborated by the benchmark study of Lamy et al. (2021) [8], which systematically evaluates several vesselness filters in the context of liver imaging. Their results show that classical Hessian-based methods such as Frangi and Sato perform well in high-contrast regions but exhibit significant performance degradation under low signal-to-noise conditions and in the presence of weak or fragmented vessels [8]. In particular, the sensitivity of these filters to parameter settings (e.g., scale range and regularization terms) leads to variability in performance across datasets. Minimal-path approaches partially address this by enforcing global optimality, but their reliance on manual seed placement limits automation and scalability in clinical workflows.

These constraints are especially problematic in infrapopliteal CTA, where small vessel calibres and pathological alterations amplify fragmentation effects. In patients with peripheral artery disease and chronic limb-threatening ischaemia, treatment planning depends on the accurate assessment of multilevel disease, distal runoff, and potential revascularisation targets [5,6]. In this setting, an artificial interruption of an infrapopliteal or pedal artery may alter the perceived availability of a distal target and reduce the clinical usability of the reconstructed vascular tree. These limitations ultimately restrict the clinical applicability of purely geometric reconstructions.

### 4.2. Deep Learning-Based Methods

Deep learning methods significantly improve voxel-wise segmentation performance by leveraging large annotated datasets to learn complex appearance features. These models are particularly effective at capturing variations in contrast and anatomy, enabling robust vessel detection even in challenging imaging conditions. Nevertheless, the results indicate that these approaches remain fundamentally limited in preserving vascular topology. Because predictions are performed at the voxel level, without explicit structural constraints, discontinuities frequently arise, especially in distal vessels or regions affected by disease. By contrast, the topology-aware deep learning methods identified in this review, such as Topo-UNet and AVDNet, represent a substantial advance over conventional architectures by embedding connectivity-oriented mechanisms, such as spatial continuity modules or topology-based refinement losses, directly within the learning process. These approaches report improved centerline-based metrics compared with conventional segmentation networks, suggesting that explicit topological supervision can partially compensate for the limitations of voxel-wise learning. The choice of evaluation metrics further exacerbates this limitation. Commonly used overlap-based measures, such as Dice score, do not adequately capture connectivity errors, allowing models to achieve high quantitative performance despite producing anatomically inconsistent segmentations. Therefore, topology-aware models achieve connectivity metrics that are improved relative to conventional deep learning approaches, while maintaining comparable Dice scores. From a clinical perspective, this distinction is important because a small segmentation gap may involve only a limited number of voxels while changing the reconstructed appearance of a vessel from patent to occluded. Such errors may impair centreline extraction, quantitative stenosis assessment, procedural planning, and the identification of distal arterial branches, despite having only a marginal effect on the global Dice score.

In parallel, other studies have focused on scaling both dataset size and diversity to develop large generalised segmentation models, commonly referred to as foundation models, such as VesselFM [47]. While these approaches have been reported to improve segmentation robustness across datasets, evidence that they explicitly improve vascular connectivity remains limited, as topological properties are not enforced at inference and connectivity gains, when reported, appear to result indirectly from improved generalization rather than from dedicated topological mechanisms. In addition, generalization across datasets remains a concern, as models may be sensitive to variations in acquisition protocols or annotation quality.

### 4.3. Hybrid Methods

Hybrid methods attempt to bridge the gap between geometric reasoning and data-driven learning by combining segmentation networks with explicit connectivity refinement strategies. As illustrated by approaches such as CorSegRec, supervised reconnection can effectively recover missing links between fragmented vessel segments and improve topological metrics beyond those of the base network. When compared with topology-aware deep learning methods identified in this review, hybrid approaches do not show a consistent advantage: some hybrid pipelines report connectivity metrics comparable to or lower than those achieved by end-to-end topology-aware networks such as Topo-UNet or AVDNet, while others, particularly graph-based reconnection methods, achieve more explicit and interpretable correction of connectivity errors. Based on the current evidence, it therefore remains uncertain whether hybrid methods systematically outperform topology-aware deep learning models, and this comparison is further limited by the heterogeneity of datasets and metrics used across studies. However, these methods introduce additional complexity, including separate training procedures and the need for dedicated reconnection annotations. Connectivity restoration should not be considered successful solely because disconnected components have been joined. Reconnection methods must also avoid anatomically implausible shortcuts, false side branches, and erroneous connections between adjacent vessels. A false connection may be as clinically misleading as a missed vessel segment, as it can create a non-existent arterial pathway or obscure the presence of a true occlusion. Therefore, future methods should evaluate both the recovery of true vascular continuity and the risk of introducing anatomically incorrect connections. Moreover, they remain dependent on the initial segmentation quality, limiting their ability to correct large-scale structural errors. Other hybrid strategies incorporate geometric priors or tracking-based refinements, but often rely on heuristics or partial user interaction, which constrains full automation. Overall, while hybrid approaches represent a meaningful step toward integrating local detection with global structure, they still fall short of providing a unified, fully automated solution that ensures both accuracy and topological consistency across diverse clinical scenarios. Furthermore, hybrid pipelines typically require sequential training of multiple components (e.g., segmentation network followed by graph-based reconnection), increasing computational and engineering overhead compared with single-stage end-to-end deep learning models, even when final inference time remains comparable.

The reviewed literature shows a clear methodological progression from local, interpretable geometric models toward data-driven approaches with increasing, though still partial, topological awareness. Hybrid strategies currently represent the most explicit attempt to reconcile accuracy with connectivity, but no approach yet offers a fully automated, generalisable solution. This trend highlights the need for standardized topology-aware benchmark datasets and consensus reporting guidelines specifically addressing vessel connectivity, which would allow more consistent comparison across future studies.

### 4.4. Limitation of the Study

This study has several limitations that should be acknowledged. First, as a scoping review, its aim was to provide a comprehensive overview of existing methodologies rather than a quantitative synthesis of performance. Accordingly, no formal meta-analysis was performed, and direct comparison of results across studies was limited by evaluation protocols and reported metrics. This review was not prospectively registered (e.g., PROSPERO). In addition, no formal quality or risk-of-bias assessment of the included studies was performed.

Second, the assessment of vessel discontinuity and connectivity preservation remains inherently challenging due to the lack of standardized definitions and dedicated evaluation criteria. Most included studies relied on overlap-based metrics, such as the Dice coefficient, which are not designed to capture topological errors. This limitation hinders consistent evaluation of the ability of different approaches to preserve vascular continuity.

Third, the literature search was restricted to selected databases and predefined keywords, which may have resulted in the omission of relevant studies, particularly those not explicitly addressing connectivity or topology despite targeting related problems. Part of the search relied on Google Scholar, whose ranking algorithm and indexing are not fully transparent, which may limit the reproducibility of the search strategy compared with structured databases such as PubMed or Embase. In addition, only articles published in English were included, introducing a potential language bias. Some included studies were published as conference proceedings rather than journal articles, and peer-review standards may vary accordingly.

Finally, although this review focuses on CTA-based segmentation, vessel breakage is a modality-agnostic issue that also affects other imaging techniques, such as MR angiography and ultrasound. Therefore, the generalisability of the present findings beyond CTA may be limited.

Future research would benefit from the development of standardized benchmarks, topology-aware evaluation metrics, and publicly available datasets to enable more robust and reproducible comparisons between methods.

## 5. Conclusions

To our knowledge, this is the first systematic review specifically focused on vessel breakage correction in CTA segmentation, providing a structured comparison of geometric, deep learning, and hybrid approaches through the lens of connectivity preservation rather than overlap-based accuracy alone. This systematic review highlights that preserving vascular continuity remains a key limitation across current vessel segmentation methods. While classical geometric approaches provide robust and interpretable tools for vessel enhancement and tracking, they are limited by their local nature and lack of automation. Deep learning methods achieve high segmentation accuracy but often fail to explicitly account for vascular topology, leading to persistent disconnections despite strong overlap-based performance.

Hybrid approaches offer a promising compromise by combining data-driven learning with geometric or graph-based constraints, enabling more explicit handling of connectivity. However, these methods remain relatively complex and are not yet standardized.

A central finding of this review is that conventional evaluation metrics, such as Dice, while valuable for assessing voxel-wise overlap, are not sufficient to reflect clinically relevant errors related to vessel breakage. Topology-aware metrics, including connectivity and centreline-based measures, are essential to properly assess segmentation quality in this context.

Future research should prioritize the development of standardized topology-aware benchmark datasets, consensus reporting guidelines for vessel connectivity assessment, and the integration of topological constraints directly into segmentation frameworks, rather than relying solely on voxel-based evaluation metrics. Moving beyond voxel-wise accuracy toward structure-aware modelling is essential for achieving reliable and clinically applicable vascular segmentation, with direct benefits for computational hemodynamic simulation, surgical planning and surveillance, automatic sizing and endovascular intervention [48,49].

## Figures and Tables

**Table 1:** Summary of deep learning vessel segmentation methods from CTA images.

Reference	Body	Description	Dataset (*n*)	Performance
Epifanov et al., 2025 [37]	Abdominal	Hybrid U-Net with gradient-based vessel refinement	LIDC-IDRI (142), AMOS	SD: 0.79, ASSD: 0.93
Ozcelik et al., 2026 [31]	Aorta	Topology-aware loss via persistent homology and Wasserstein distance	AortaSeg24 (24)	P: 0.88, Rec: 0.87, F: 0.87
Zhao et al., 2025 [32]	Aorta	Scale-aware representation with morphology-guided attention	ImageTBAD (100)	CL: 95%
Iltaf et al., 2025 [38]	Aorta	VesselFAM: SAM-based model with atrous attention	AVT (56), ImageT (100)	Dice: 0.72, ASSD: 0.93
Liu et al., 2025 [36]	Cerebral	DTUNet: dual-branch topology-aware UNet	private (50)	OV: 0.79, ASCD: 1.05
Wu et al., 2026 [33]	Coronary & cerebral	FlowAxis: continuous vessel representation with adaptive keypoints	ASOCA (40), ImageCAS	clDice: 0.90, HD: 6.0
Ye et al., 2025 [35]	Coronary	VSR-Net: rupture rehabilitation using curvilinear clustering	ImageCAS (1000)	Dice: 0.89, Jacc: 0.79
Acebes et al., 2024 [29]	Coronary	clCE loss combining cross-entropy with clDice topology constraints	ASOCA (40)	Dice: 0.80, clDice: 0.79
Chen et al., 2026 [28]	Coronary	CLGeoDice: skeleton matching via continuous linear distance metric	private (160)	Dice: 0.87, clDice: 0.88
Liu et al., 2026 [34]	Coronary	TopoVST: skeleton extraction via multi-scale sphere graphs and GNNs	ASOCA (40), AortaSeg24 (24)	ASOCA(OV: 0.87, B0: 2.7) AortaSeg24(OV: 0.95, B0: 1.1)
Guzzi et al., 2026 [4]	Coronary, hepatic, lower limbs	Multi-territory vessel segmentation for PAD analysis (PADSET)	private (176)	Dice: 0.94, clDice: 0.88
Yao et al., 2023 [30]	Head & neck	TaG-Net: voxel segmentation with centerline-based vascular graph topology	private (401)	Dice: 0.92, HD95: 3.6

P: Precision, Rec: Recall, F: F-score, CL: Centerline overlap, OV: Overlap, Jacc: Jaccard, ASCD: average symmetric centerline distance, SD: Surface Dice, ASSD: Average Symmetric Surface Distance, B0: Betti number of order 0, HD: 95% Hausdorff Distance.

**Table 2:** Summary of hybrid vessel segmentation methods from CTA images.

Reference	Body	Description	Dataset (*n*)	Performance
Qiu et al., 2025 [42]	Coronary	CorSegRec: graph-based reconnection enforcing full arterial tree connectivity	ASOCA (40)	Dice: 0.88, HD: 1.07
Alirr et al., 2024 [40]	Coronary	Vesselness enhancement, ROI extraction, and ResUNet segmentation	ASOCA (40), MM-WHS (60)	Dice: 0.87, Rec: 0.88
Park et al., 2026 [41]	Coronary	U-Net with gradient-based contour detection for boundary refinement	ASOCA (40)	Dice: 0.82, CL:8.9
Wolterink et al., 2019 [1]	Coronary	GCN predicting vertex positions of tubular surface meshes	Private (87)	Dice: 0.73, HD: 1.86
Wang et al., 2024 [45]	Coronary	AVDNet: joint artery–vein segmentation with topology refinement (TVRN) and IDD loss	ASOCA (40)	Dice: 0.88, cl-rec: 3.6
Survarachakan et al., 2021 [39]	Hepatic	Vesselness filtering, gamma correction, and feature fusion prior to CNN segmentation	Private (57)	Dice: 0.82, ASSD: 1.0
Esposito et al., 2025 [44]	Hepatic	VesselSDF: neural signed distance function for continuous vascular surface modeling	3Dircadb (20)	Dice: 0.86 IoU: 0.82
Guo et al., 2020 [43]	Hepatic	Multi-stage pipeline: preprocessing, 3D graph cuts, centerline extraction, completion	3Dircadb (20)	Acc: 0.98 Rec: 0.66
Liu et al., 2026 [46]	Pulmonary	Topo-UNet: multi-task topology-aware UNet with BS-ConvLSTM and auxiliary refinement	ISICDM 2020 (43)	Dice: 0.91, IoU: 0.83

Rec: Recall, cl-rec: Centerline recall, IoU: Intersection over Union, Acc: Accuracy, ASSD: Average Symmetric Surface Distance, HD: 95% Hausdorff Distance, CL: Centerline overlap.

## Data Availability

No new data were created or analyzed in this study. Data sharing is not applicable to this article.

## Supplementary Materials

The following supporting information can be downloaded at https://www.mdpi.com/article/10.3390/jcm15156077/s1. Table S1: PRISMA 2020 Checklist.

## Abbreviations

The following abbreviations are used in this manuscript:

Acc	Accuracy
AUC	Area Under the Curve
AI	Artificial Intelligence
CTA	Computed Tomography Angiography
CCR	Connected Component Ratio
MRA	Magnetic Resonance Angiography
PAD	Peripheral Artery Disease
TP	Topology Precision
TS	Topology Sensitivity
ASCD	Average Symmetric Centerline Distance
CNN	Convolutional Neural Network
DL	Deep Learning
ML	Machine Learning
CT	Computed Tomography
MR	Magnetic Resonance
DSC	Dice Similarity Coefficient
HD	Hausdorff Distance
FC	Fuzzy Connectedness
FP	False Positive
FN	False Negative
TP	True Positive
